# Protective efficacy of a candidate live attenuated vaccine derived from the SD-R strain of lineage 1 porcine reproductive and respiratory syndrome virus against a lethal challenge with HP-PRRSV HuN4 in piglets

**DOI:** 10.1128/spectrum.01984-23

**Published:** 2023-10-11

**Authors:** Hongliang Zhang, Chao Li, Hu Xu, Bangjun Gong, Wansheng Li, Zhenyang Guo, Lirun Xiang, Qi Sun, Jing Zhao, Jinmei Peng, Qian Wang, Guohui Zhou, Yan-Dong Tang, Tongqing An, Xue-Hui Cai, Zhi-Jun Tian

**Affiliations:** 1 State Key Laboratory of Veterinary Biotechnology, Harbin Veterinary Research Institute, Chinese Academy of Agricultural Sciences, Harbin, China; Oklahoma State University College of Veterinary Medicine, Stillwater, Oklahoma, USA

**Keywords:** lineage 1, attenuated vaccine, SD-R, HP-PRRSV HuN4, cross-protection efficacy

## Abstract

Porcine reproductive and respiratory syndrome virus (PRRSV) is an important pathogen endangering the swine industry worldwide. Our previous study confirmed that the live attenuated vaccine SD-R strain could provide broad cross-protection against homologous and heterologous NADC30-like viruses. However, it is unclear whether SD-R can provide protection for piglets against HP-PRRSV. In the present study, 4-week-old PRRSV-free piglets were vaccinated intramuscularly with 10^6.2^ TCID_50_ of SD-R and then challenged intramuscularly (2 mL) and intranasally (2 mL) with HP-PRRSV HuN4 (1 × 10^5.0^ TCID_50_/mL). The results showed that two of five piglets died in the HuN4 challenge group and five of five immunized piglets survived in the immune challenge group. Five piglets in the HuN4 challenge group had a fever for 6–12 days and showed obvious clinical symptoms, but 3 of 5 piglets had a fever for only 1 day at 5 days post inoculation, and only 1 of the 5 piglets in the immune challenge group had a cough. Compared to challenged control piglets, vaccinated piglets had significantly improved daily weight gain and obviously alleviated pathological lesions. Furthermore, piglets immunized with SD-R had reduced thymus atrophy caused by HP-PRRSV HuN4. These results demonstrate that the lineage 1 PRRSV attenuated vaccine SD-R strain provides protection against lethal HP-PRRSV HuN4 challenge. As such, it is a promising vaccine candidate for use against PRRSV infection.

**IMPORTANCE:**

Both highly pathogenic porcine reproductive and respiratory syndrome virus (HP-PRRSV) and NADC30-like PRRSV have caused tremendous economic losses to the Chinese pig industry. In this study, a good challenge model was established to evaluate the protection afforded by the candidate SD-R vaccine against infection with a representative HP-PRRSV strain (HuN4). The control piglets in the challenge experiment displayed obvious clinical symptoms of PRRSV infection, with a mortality rate up to 40%. In contrast, all the piglets in the vaccinated challenged group survived, and only some pigs had transient fever. The daily gain of SD-R immunized group piglets was significantly increased, and the pathological changes were significantly reduced. In addition, the viral replication levels in the serum of the immunized group were significantly lower than those of the challenged control group. The live attenuated vaccine SD-R strain can provide protection against HP-PRRSV challenge, indicating that the SD-R strain is a promising vaccine candidate for use in the swine industry.

## INTRODUCTION

Porcine reproductive and respiratory syndrome (PRRS) is one of the most important diseases causing enormous economic losses to the global swine industry. PRRS virus (PRRSV), the aetiological agent of PRRS, is characterized by fast mutation, complex recombination, and large differences in pathogenicity (1). PRRSVs can be divided into two distinct species, Betaarterivirus suid 1 (PRRSV-1) and Betaarterivirus suid 2 (PRRSV-2) (ICTV2021). PRRSV-1 is divided into three or four subtypes {subtype 1 [subtype 1 (Global)], subtype 2 [subtype I (Russia) and subtype II], and subtype 3 [subtype III]} (2 – 6), and PRRSV-2 is divided into nine lineages (lineage 1-lineage 9) (6). Since 1995, subtype I (global) of PRRSV-1 and lineage 1, lineage 3, lineage 5, and lineage 8 of PRRSV-2 have coexisted in China (7). Currently, NADC30-like PRRSV and NADC34-like PRRSV in lineage 1 have become the major endemic strains in China (7 – 12). However, existing PRRSV vaccines provide only limited protection for piglets against lineage 1 PRRSV (13 – 20).

In mainland China, the first PRRSV strain, named CH-1a, belongs to sublineage 8.7 and was first reported in 1996 (21). In 2006, a highly pathogenic PRRSV (HP-PRRSV) containing a discontinuous deletion of 1 + 29 aa in the NSP2 protein caused an outbreak in China that was characterized by high morbidity and mortality (22 – 26). HP-PRRSV causes large numbers of pig deaths and tremendous economic losses in China and some pig-raising countries in Southeast Asia (24, 27, 28). The HP-PRRSV HuN4 strain was isolated and caused 100% morbidity and 40%–100% mortality in piglets (26). Subsequently, HP-PRRSV vaccines (JXA1-P80, HuN4-F112, TJM-92, and GDr180) were developed and applied throughout the country (29 – 31).

PRRSV, characterized by its mutability, produces some novel variants causing outbreaks or recurrent outbreaks in previously vaccinated or unvaccinated swine herds. Heterologous cross-protection by most PRRS vaccines is an obvious deficiency and is also the focus of veterinary researchers. In a previous study, we developed an attenuated lineage 1 PRRSV vaccine (SD-R) that provides safe and effective protection against homologous NADC30-like PRRSV and heterologous NADC30-like PRRSV challenges (32). Furthermore, SD-R could provide effective protection against NADC34-like PRRSV (unpublished data). However, the protective efficacy of SD-R against lethal HP-PRRSV challenge in piglets is unknown.

## MATERIALS AND METHODS

### Viruses and animals

The candidate live attenuated vaccine SD-R strain was attenuated and stored in our laboratory. The HP-PRRSV HuN4 strain was isolated in 2006 and is maintained in our laboratory. Thirteen 28-day-old confirmed PRRSV-free piglets (antigens of PRRSV, ASFV, CSFV, and PRV were detected using PCR or RT-PCR; antibodies against PRRSV, ASFV, CSFV, and PRV were detected using commercial ELISA kits) were obtained from a PRRS-free farm in Harbin.

### Phylogenetic analysis

All sequences were aligned using MAFFT version 7 (33) with default parameters and manually adjusted in MEGA6 (34). Deduced amino acid sequences were aligned with ClustalW with Lasergene software. Phylogenetic trees based on the whole genome were constructed in MEGA 6.0 using the neighbor-joining method with 1,000 bootstrap replicates. The trees were annotated and modified using Evolview (version 2.0) (35).

### Evaluation of the immunoprotective effect of SD-R against the HP-PRRSV HuN4 strain

Thirteen PRRSV-free piglets were randomly (the piglets were mixed together before the group assignment) divided into three groups (Groups A, B, and C) (Table 1). Five piglets in group A were used for immunization and inoculation. Five piglets in group B were used for inoculation. The other three piglets in group C were used as negative controls. The piglets in group A were inoculated intramuscularly (2 mL) with 10^6.2^ TCID_50_/mL SD-R. At 28 days post vaccination (dpv), the piglets in groups A and B were infected intramuscularly (2 mL) and intranasally (2 mL), respectively, with fifth-passage HuN4 (1 × 10^5.0^ TCID_50_/mL). The animals were maintained in individual biosafety rooms. Clinical signs and rectal temperatures were recorded daily. The body weights of the piglets were measured weekly. Blood samples were periodically collected from individual piglets and tested for viremia. All of the piglets were euthanized at 21 days post inoculation (dpi). Ten tissue samples were obtained from the hearts, livers, spleens, lungs, kidneys, lymph nodes, tonsils, small intestines, bladders, and stomachs for viral detection by TaqMan-based real-time fluorescence quantitative RT-PCR (36).

**TABLE 1:** Group information for animal experiments

Group	Corresponding group	Number of animals	Vaccination	Challenge
A	SD-R vaccine-treated and HuN4-challenged group	5 (091; 092; 093; 094; 035)	1 × 10^6.2^ TCID_50_ per pig (SD-R)	4 × 10^5.0^ TCID_50_ per pig (HuN4)
B	HuN4-challenged group	5 (062; 063; 064; 065; 066)	DMEM	
C	Negative control group	3 (067; 068; 069)		DMEM

### Serological examination

Serum samples were collected at 0, 7, 14, 21, and 28 dpv and at 3, 5, 7, 10, 14, and 21 dpi. PRRSV-specific antibodies were quantified using a commercial ELISA kit (JNT, Beijing, China) according to the manufacturer’s instructions. The PRRSV-specific antibody titer is reported as the S/P ratio, and the serum samples were considered positive if the S/P ratio was ≥0.4.

To perform serum neutralization (SN) assays, all sera collected at 28 dpv and 21 dpi were heat-inactivated at 56°C for 30 min. Subsequently, serial twofold dilutions of each serum sample were prepared using DMEM as the diluent. Suspensions containing 100 TCID_50_ of PRRSV per 100 µL were then prepared, and 100 µL of the suspension was added to each serum dilution. The serum-viral mixtures were incubated for 1 h at 37°C in a water bath. Then, the mixtures were dispensed onto MARC-145 cells in 96-well plates. The plates were further incubated at 37°C in a humidified atmosphere with 5% CO_2_ for 7 days. Duplicate samples were analyzed for CPE (cytopathic effect) daily. The neutralization titer of the serum was calculated using the Reed-Muench method. Three independent tests were performed for each serum sample.

### Viremia and viral loads in tissue assessment

To determine the duration of viremia and viral loads in different tissues after treatment with the SD-R vaccine strain, serum samples collected at 0, 7, 14, 21, and 28 dpv and 3, 5, 7, 10, 14, and 21 dpi and 10 tissues of all the piglets were used to detect the RNA copy number of PRRSV by TaqMan-based real-time fluorescence quantitative RT-PCR (36).

### Histological examination

At necropsy, the lungs and lymph nodes were harvested and examined for histopathology following hematoxylin and eosin (H&E) staining as previously described (37).

### Statistical analysis

Significant differences between two groups were determined using a *t* test (and nonparametric tests) in GraphPad 5.0 (San Diego, CA, USA). The level of significance was set at *P* < 0.05.

## RESULTS

### Genomic characteristics of NADC30-like PRRSV SD-R and HP-PRRSV HuN4

**Fig 1:**
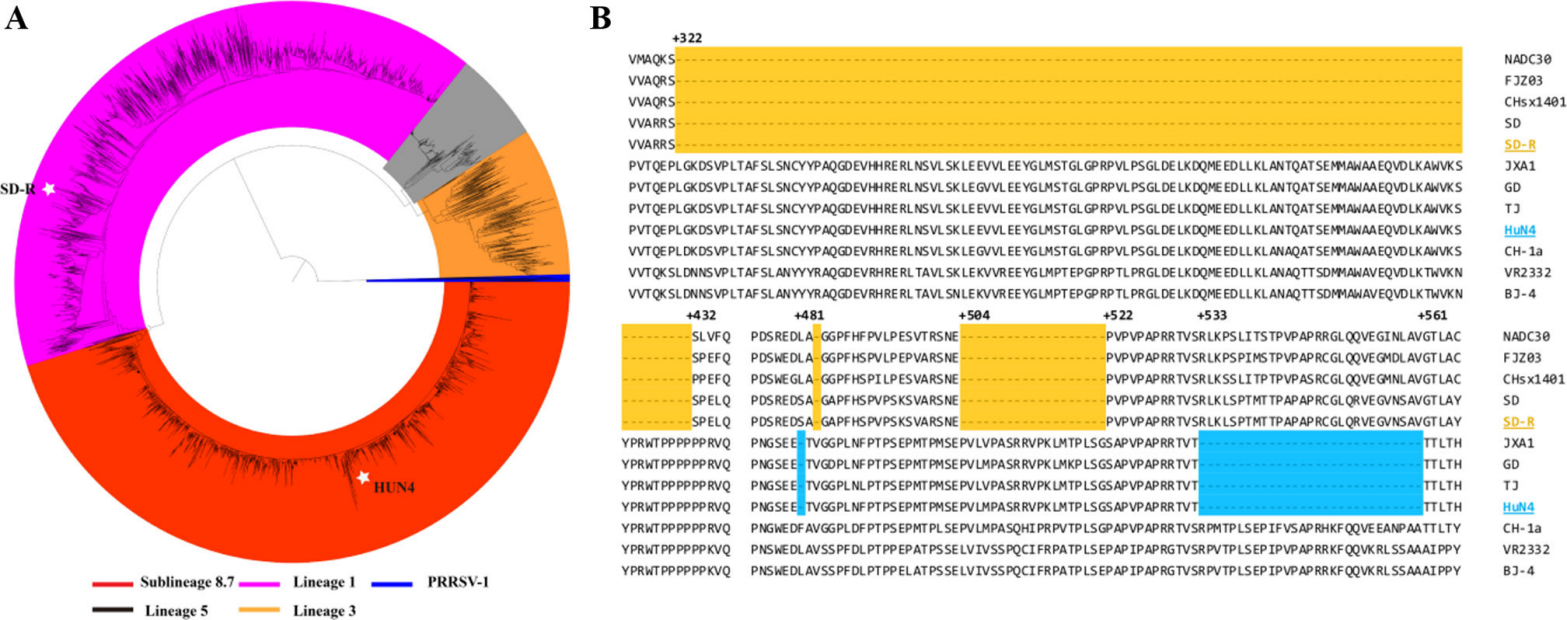
Phylogenetic analyses and NSP2-deduced amino acid alignment of the SD-R and HuN4 strains. (**A**) Phylogenetic tree of SD-R and HuN4 based on all the ORF5 sequences of PRRSV. The five lines with different colors represent the five types of PRRSVs. Both SD-R and HuN4 are labeled with white pentagons. (**B**) The NSP2 deletion pattern in SD-R and HuN4: 131-aa discontinuous deletions are labeled with a light-yellow background; 30-aa discontinuous deletions are labeled with a light blue background.

To clarify the relationship between NADC30-like PRRSV SD-R and HP-PRRSV HuN4, the ORF5 sequences of 5,477 PRRSV isolates from China were used to construct a phylogenetic tree. Phylogenetic analysis showed that five subgenotype PRRSVs coexisted in China, including PRRSV-1 subtype I (Global) and PRRSV-2 lineages 1, 3, 5, and 8 (Fig. 1A). SD-R belongs to lineage 1 (1.8), but HuN4 belongs to lineage 8 (8.7) (Fig. 1A). The deduced amino acid alignment of NSP2 showed that SD-R has a 131 aa (111 + 1 + 19) discontinuous deletion and that HuN4 has a 30 aa (1 + 29) discontinuous deletion (Fig. 1B). The genomic nucleotide similarity between SD-R and HuN4 was 83.5%, and the nucleotide similarity and deduced aa similarity among different genes were 75.2%–93.3% and 70.8%–96.7%, respectively (Table 2).

**TABLE 2:** The nucleotide and deduced amino acid similarity between SD-R and HuN4

Gene	Nucleotide similarity (%)	Deduced amino acid similarity (%)	Gene	Nucleotide similarity (%)	Deduced amino acid similarity (%)
Whole genome	83.5	/	Nsp9	86.6	96.7
5′UTR	89.4	/	Nsp10	84.1	95.0
3′UTR	93.3	/	Nsp11	89.8	95.1
Nsp1α	87.2	94.4	Nsp12	87.1	94.8
Nsp1β	80.0	75.2	ORF2a	87.8	88.3
Nsp2	75.2	70.8	ORF2b	90.5	90.5
Nsp3	81.3	89.1	ORF3	86.7	83.5
Nsp4	85.0	93.1	ORF4	85.8	86.6
Nsp5	87.5	93.5	ORF5	86.5	86.9
Nsp6	91.7	93.8	ORF5a	87.0	82.6
Nsp7α	83.0	91.3	ORF6	89.4	93.1
Nsp7β	77.3	74.5	ORF7	89.2	90.3
Nsp8	87.4	93.3	N		

### Clinical reactions after immunization and challenge

**Fig 2:**
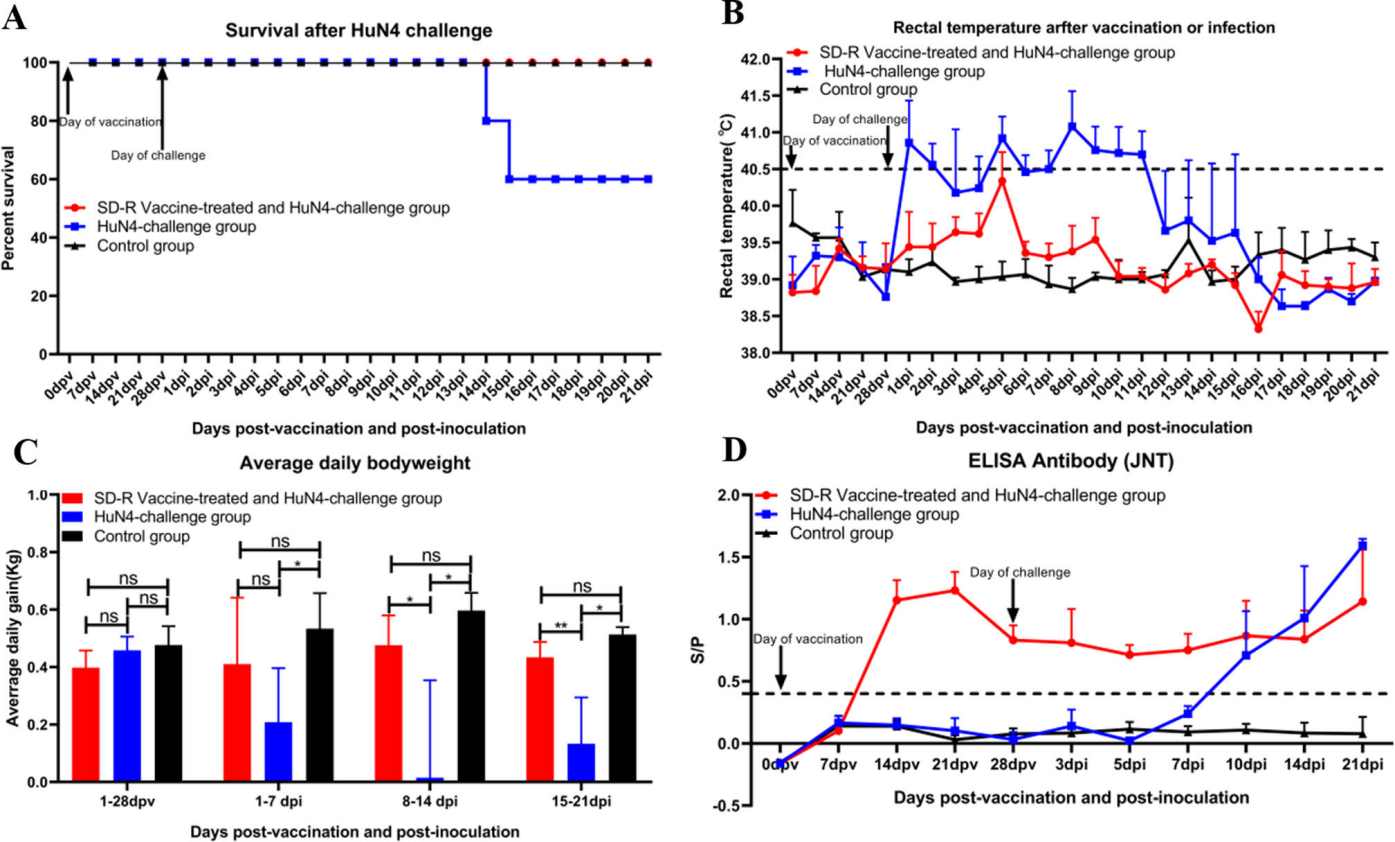
Survival, rectal temperature, average daily bodyweight, and anti-PRRSV antibody levels after SD-R immunization and challenge with the HP-PRRSV HuN4 strain. (**A**) Survival after HuN4 challenge. (**B**) Rectal temperatures after SD-R immunization and challenge with the HuN4 strain. (**C**) Average daily body weight after SD-R immunization and challenge with the HuN4 strain. (**D**) Anti-PRRSV antibody levels after SD-R immunization and challenge with the HuN4 strain. Tree groups are labeled with red, blue, and black.

After SD-R immunization, none of the piglets in group A showed any clinical signs of PRRS in contrast to those in group B and group C. After HuN4 challenge, two piglets in group B died at 14 and 15 dpi, but all the piglets in groups A and C survived (Fig. 2A). All the piglets in group B displayed various disease manifestations, including high fever (≥41.0) (Fig. 2B), listlessness, anorexia and emaciation, lying down, and cough. Three piglets had a fever for only 1 day, at 5 dpi (Fig. 2B), and only 1 piglet in group A showed a cough. During the study, the piglets in group C had no clinical signs of disease. After HuN4 challenge, compared to the piglets in groups A and C, the piglets in group B gained less body weight (*P* < 0.05) from 1 to 7, 8 to 14, and 15 to 21 dpi (Fig. 2C).

### Antibody responses in immunized or challenged piglets

The antibody response determined via ELISA showed that all the immunized piglets in group A were seroconverted at 14 dpv (Fig. 2D). A total of 4 of 5 piglets in group B were seroconverted by 10 dpi, and the remaining piglets were seroconverted by 14 dpi (Fig. 2D). No PRRSV-specific antibodies were detected in the control piglets prior to challenge (Fig. 2D). The antibody responses of the piglets in group C were negative throughout the study (Fig. 2D).

### Gross pathological and histopathological changes

**Fig 3:**
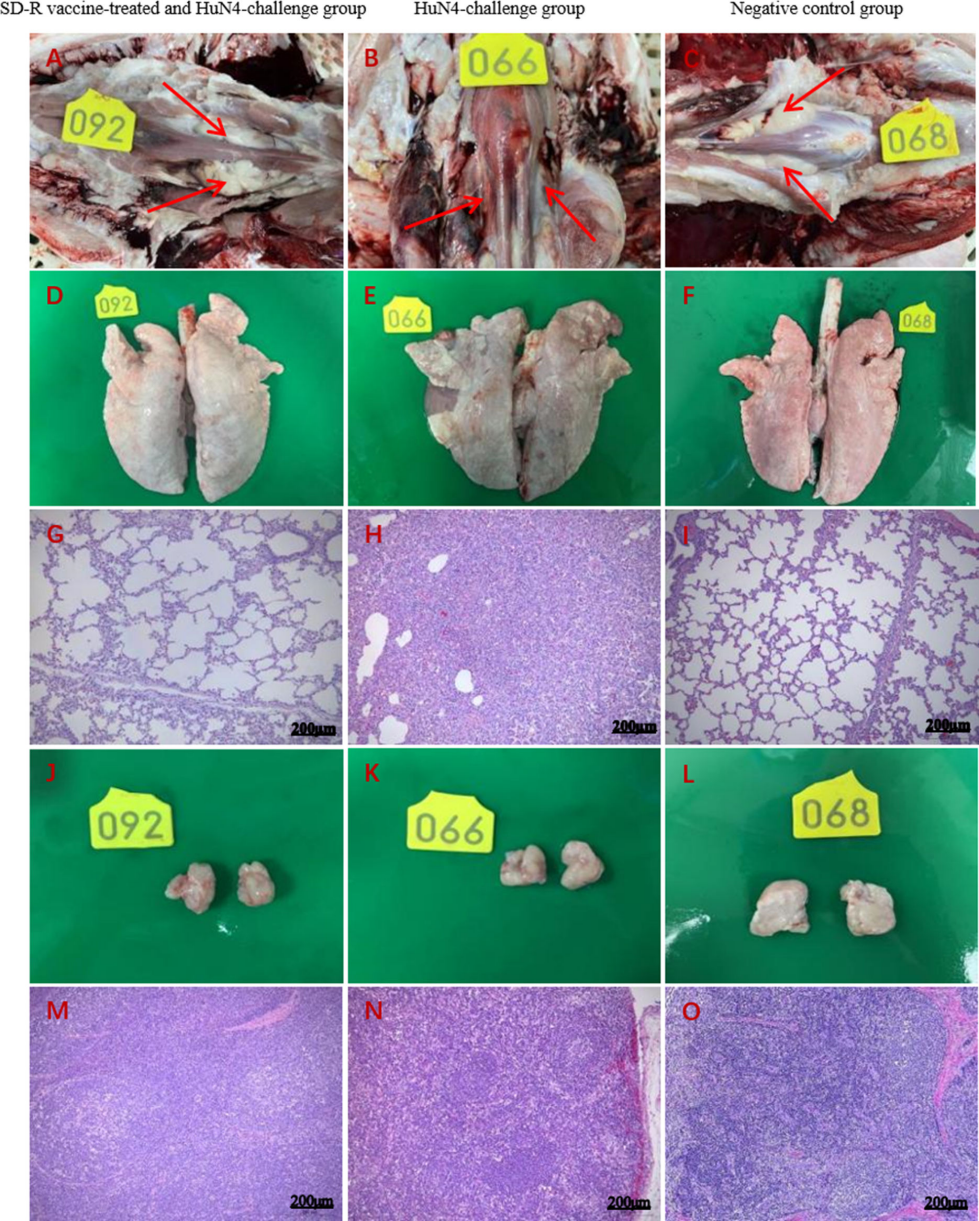
Gross and histological lesions of lungs and lymph nodes after SD-R immunization and challenge with the HP-PRRSV HuN4 strain. In contrast with the piglets in the immunized and challenge group (**A, D, and J**) and the negative group (**C, F, and L**), the piglets in the HuN4 challenge group showed lesions typical of PRRS, such as thymus atrophy (**B**) and consolidation in the lungs (**E**). Compared with those in the negative control group (**I, O**), some piglets in the immunized and challenge groups (**G, M**) showed milder infiltration of inflammatory cells in the lungs and slightly decreased levels of lymphocytes. All the piglets in the HuN4-challenged group showed extensive infiltration of inflammatory cells, loss of alveolar structure over large areas, airway obstruction in the lungs (**H**), and decreased lymphocyte and medullary bleeding in the lymph nodes (**N**).

All piglets were euthanized and dissected at 21 dpi (after HuN4 challenge). In contrast with the piglets in groups A (Fig. 3A, D, and J) and C (Fig. 3C, F, and L), the piglets in group B showed lesions typical of PRRS, such as thymus atrophy (Fig. 3B) and consolidation in the lungs (Fig. 3E). Histopathology revealed extensive infiltration of inflammatory cells, loss of alveolar structure over large areas, airway obstruction in the lungs (Fig. 3H), and decreased lymphocyte and medullary bleeding in the lymph nodes (Fig. 3N) in group B compared with those in the negative control groups (Fig. 3I and O). Notably, some piglets in group A showed milder infiltration of inflammatory cells in the lungs (2 of 5) and slightly decreased levels of lymphocytes (3 of 5) (Fig. 3G and M).

### Comparison of viremia and viral tissue distribution between the immunized-challenge group and the challenge group

**Fig 4:**
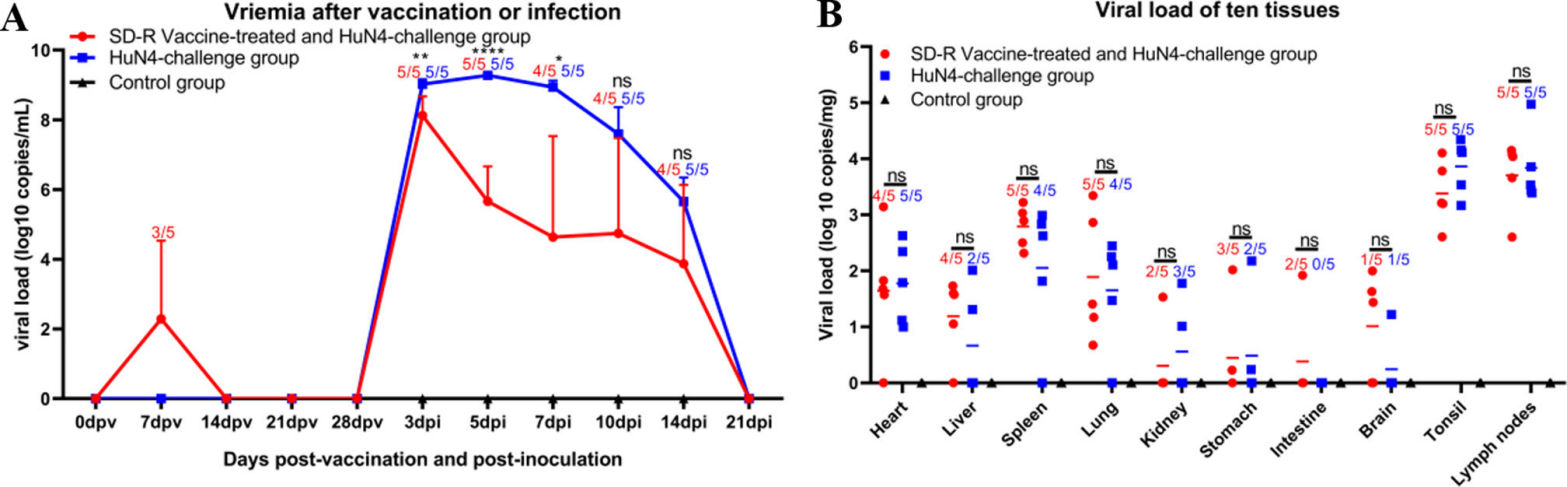
Viremia (**A**) and viral loads in 10 tissues (**B**) after SD-R immunization and challenge with the HP-PRRSV HuN4 strain. PRRSV viral RNA in sera and tissues was quantified by qPCR. An asterisk (*) indicates a significant difference between the immunized and challenge groups and the HuN4 challenge group (ns, *P* > 0.5; *P < 0.05; **P < 0.01; ****P < 0.0001). The numbers represent the number of piglets with viral loads and the number of piglets in the group. The three groups are labeled with red, blue, and black, respectively.

To evaluate the difference in viremia and distribution in 10 tissues among the different groups, serum samples from 0, 7, 14, 21, and 28 dpv; serum samples from 3, 5, 7, 10, 14, and 21 dpi; and 10 organ tissues were evaluated using real-time PCR. The results showed that 3 of the 5 piglets in group A had low levels of viremia at 7 dpv (Fig. 4A). The RNA copy numbers of the serum samples from group B reached their highest levels at 3 dpi and then gradually declined after 10 dpi (Fig. 4A). The viremia levels at 3–7 dpi in group A were significantly lower than those in group B (Fig. 4A). The viral loads of the 10 tissues were not significantly different between group A and group B (Fig. 4B).

### Serum-neutralizing antibody detection

To explore the role of SN Ab in conferring protection, an SN assay was performed using serum samples collected at 28 dpv and 21 dpi from all pigs. CPE was found in all the control wells at 3 days after cell inoculation (100 TCID_50_/well). In Group A, 5/5 and 1/5 pigs immunized with SD-R (28 dpv) induced homologous SN Ab on days 3 and 7 after cell inoculation, respectively. In addition, all pigs immunized with SD-R (21 dpi) induced homologous SN Ab on days 3 and 7 after cell inoculation (Table 3). In the SN assay against the HuN4 strain, at 28 dpv, neither group A nor group B developed SN Ab against the HuN4 strain either (Table 4). No neutralization titers were detected in group B pigs at 21 dpi. In contrast, serum from group A pigs at 21 dpi could delay the appearance of CPE on the third day of the SN assay, and 3 pigs were able to completely neutralize the HuN4 strain on day 7 (Table 4). No SN Ab was found in the challenge control group and negative control group.

**TABLE 3:** Serum neutralization titer of three different group against SD-R strain

Vaccinated challenged group	Serum neutralization titer		Challenged group	Serum neutralization titer		Negative control group	Serum neutralization titer	
	3 days	7 days		3 days	7 days		3 days	7 days
091 28 dpv	5.62	<1:4	062 28 dpv	<1:4	<1:4	067 28 dpv	<1:4	<1:4
092 28 dpv	5.62	<1:4	063 28 dpv	<1:4	<1:4	068 28 dpv	<1:4	<1:4
093 28 dpv	6.32	<1:4	064 28 dpv	<1:4	<1:4	069 28 dpv	<1:4	<1:4
094 28 dpv	5.62	<1:4	065 28 dpv	<1:4	<1:4	067 21 dpi	<1:4	<1:4
035 28 dpv	11.34	4	066 28 dpv	<1:4	<1:4	068 21 dpi	<1:4	<1:4
091 21 dpi	16	16	062 21 dpi	<1:4	<1:4	069 21 dpi	<1:4	<1:4
092 21 dpi	22.68	4	063 21 dpi	<1:4	<1:4			
093 21 dpi	27.12	16	064 21 dpi	<1:4	<1:4			
094 21 dpi	32	32	065 21 dpi	<1:4	<1:4			
035 21 dpi	40	32	066 21 dpi	<1:4	<1:4			

**TABLE 4:** Serum neutralization titer of three different group against HuN4 strain

Vaccinated challenged group	Serum neutralization titer		Challenged group	Serum neutralization titer		Negative control group	Serum neutralization titer	
	3 days	7 days		3 days	7 days		3 days	7 days
091 28 dpv	<1:4	<1:4	062 28 dpv	<1:4	<1:4	067 28 dpv	<1:4	<1:4
092 28 dpv	<1:4	<1:4	063 28 dpv	<1:4	<1:4	068 28 dpv	<1:4	<1:4
093 28 dpv	<1:4	<1:4	064 28 dpv	<1:4	<1:4	069 28 dpv	<1:4	<1:4
094 28 dpv	<1:4	<1:4	065 28 dpv	<1:4	<1:4	067 21 dpi	<1:4	<1:4
035 28 dpv	<1:4	<1:4	066 28 dpv	<1:4	<1:4	068 21 dpi	<1:4	<1:4
091 21 dpi	10	10	062 21 dpi	<1:4	<1:4	069 21 dpi	<1:4	<1:4
092 21 dpi	10	<1:4	063 21 dpi	<1:4	<1:4			
093 21 dpi	16	6.32	064 21 dpi	<1:4	<1:4			
094 21 dpi	5	<1:4	065 21 dpi	<1:4	<1:4			
035 21 dpi	27.12	16	066 21 dpi	8	<1:4			

## DISCUSSION

Both PRRSV-1 and PRRSV-2 are important diseases threatening the normal development of the swine industry (38). Two genotypes (PRRSV-1 and PRRSV-2), including six subgenotypes [subtype I (Global) and lineages 1.5, 1.8, 3.5, 5.1, and 8.7 (C-PRRSV and HP-PRRSV)], coexisted in mainland China (7, 9). Among them, lineages 1.8 (NADC30-like PRRSV) and 8.7 (HP-PRRSV) caused tremendous economic losses to the Chinese pig industry (23, 39). Two types of PRRSV vaccines (lineage 5: RespPRRS MLV/Ingelvac PRRS MLV; lineage 8: CH-1a, CH-1R, JXA1-P80, HuN4-F112, TJM-92, GDr180, PC) have been extensively used in China (15, 29 – 31, 40, 41).

Currently, PRRSV vaccines used in China are all derived from two lineages: lineage 5 (RespPRRS MLV/Ingelvac PRRS MLV) and lineage 8 (CH-1a, CH-1R, JXA1-P80, HuN4-F112, TJM-92, GDr180, PC). The pathogenicity of NADC30-like PRRSV in piglets varies from low to high (31, 39, 42 – 47), but most show moderate pathogenicity (13, 43, 48 – 53). None of the commercial PRRSV vaccines provides complete protection for piglets against NADC30-like PRRSV challenge (13, 14, 16 – 19, 31, 54, 55). The pathogenicity of most HP-PRRSVs is high in piglets (25, 26, 55 – 57). HP-PRRSV vaccines (JXA1-P80, HuN4-F112, TJM-92, and GDr180) could provide complete clinical protection to piglets (15, 29, 31).

Since 2016, NADC30-like PRRSV has become the major endemic strain in China (9, 39). A candidate live attenuated vaccine (SD-R strain) against NADC30-like PRRSV was developed by our laboratory, which has higher safety, provides complete clinical protection for piglets against homologous and heterologous NADC30-like PRRSVs (32) and NADC34-like PRRSV (unpublished), and significantly improves daily weight gain and decreases viremia (32). Thus, SD-R could provide good protection against lineage 1 branch PRRSV. However, it is unknown whether SD-R could provide protection for piglets against a lethal HP-PRRSV challenge. Therefore, in this study, we systematically evaluated the protective efficacy of SD-R against an HP-PRRSV HuN4 strain.

Within 28 days after immunization, none of the immunized piglets showed any clinical signs of PRRS, and all immunized piglets were seroconverted at 14 dpv. There was no significant difference between immunized piglets and negative control piglets in daily weight gain. Only 3 of the 5 immunized piglets had low levels of viremia at 7 dpv, which also further demonstrated that SD-R had a higher safety. After HuN4 challenge, 2 nonimmunized piglets died at 14 and 15 dpi, but all the immunized piglets survived, which means that SD-R could prevent a lethal attack by the HP-PRRSV HuN4 strain. All the nonimmunized piglets displayed various disease manifestations, but only three immunized piglets had a fever for 1 day at 5 dpi, and only 1 immunized piglet showed a cough. Furthermore, piglets immunized with SD-R had improved daily weight gain during the duration of the HuN4 challenge. The above results demonstrated that SD-R could provide at least 80% clinical protection for piglets against HP-PRRSV HuN4 challenge.

Previous research has shown that serum-neutralizing antibodies (SN Abs) play a crucial role in the development of protective immunity against PRRSV (58, 59). Moreover, SN Abs are usually specific for the vaccine strain (homologous), with lower/no titers of cross-neutralizing (heterologous) antibodies (60, 61). In this study, we did not detect neutralizing titers against the HuN4 strain in any 28 dpv serum. Interestingly, we found that the group A pig serum at 21 dpi could delay or completely prevent the HuN4 strain from infecting Marc-145 cells. Thus, although neutralizing titers were not detected at 28 dpv, prophylactic immunization with the SD-R vaccine could help animals generate heterologous neutralizing antibodies in the later stages, reducing the impact of the HuN4 strain on pigs.

Except for death and obvious clinical manifestation, after HP-PRRSV HuN4 challenge, obvious pathological changes, especially thymus atrophy, tissue pathological changes, and high viremia, were observed in the challenged pigs, as observed in previous studies (62 – 67). However, immunized piglets of SD-R could prevent visible pathological damage caused by HuN4, especially preventing thymus atrophy and consolidation in the lungs. Furthermore, piglets immunized with SD-R had significantly alleviated histopathological damage. In addition, piglets immunized with SD-R had significantly reduced viremia, and SD-R helped the pigs produce heterologous neutralizing antibodies in the later stages. At the genus level, SD-R could provide good protection for piglets against lineage 1 and lineage 8 branch PRRSVs, but the protective efficacy against homologous PRRSV was better than that against heterologous PRRSVs. Existing commercial vaccines cannot provide effective protection against other branches of PRRSV strains (13, 19, 32, 41), but SD-R exhibits better cross-protection against different branches of PRRSV strains. Therefore, the NADC30-like PRRSV SD-R strain is a promising vaccine candidate against different PRRSVs. However, the cross-protection mechanism of SD-R needs further study.

### Conclusions

The HP-PRRSV HuN4 strain is highly pathogenic in piglets. The lineage 1 PRRSV candidate vaccine strain SD-R produced low-level viremia in some piglets at some time points after immunization. SD-R provided effective protection against lethal attack by the HuN4 strain in piglets, prevented thymus atrophy and consolidation in the lungs, significantly improved daily weight gain and other clinical manifestations, and significantly decreased viremia. SD-R is a promising vaccine candidate against PRRSV.
